# Against the tide: the role of bacterial adhesion in host colonization

**DOI:** 10.1042/BST20160186

**Published:** 2016-12-02

**Authors:** Daniel Henry Stones, Anne Marie Krachler

**Affiliations:** Institute of Microbiology and Infection, School of Biosciences, University of Birmingham, Edgbaston, Birmingham B15 2TT, U.K.

**Keywords:** bacterial adhesion, cell adhesion, host–microbe interactions, microbiology

## Abstract

Evolving under the constant exposure to an abundance of diverse microbial life, the human body has developed many ways of defining the boundaries between self and non-self. Many physical and immunological barriers to microbial invasion exist, and yet bacteria have found a multitude of ways to overcome these, initiate interactions with and colonize the human host. Adhesion to host cells and tissues is a key feature allowing bacteria to persist in an environment under constant flux and to initiate transient or permanent symbioses with the host. This review discusses reasons why adhesion is such a seemingly indispensable requirement for bacteria–host interactions, and whether bacteria can bypass the need to adhere and still persist. It further outlines open questions about the role of adhesion in bacterial colonization and persistence within the host.

## Introduction

Bacteria have evolved an abundance of mechanisms to engage with host cells, and manipulate their cellular signaling programmes to facilitate colonization. Most, if not all of these, strictly depend on bacterial adhesion to host cells: upon initial contact with host cells, bacteria sense the change in physicochemical properties in their environment (i.e. surface sensing) and can dramatically alter their physiology to respond and adapt. Changes in response to surface engagement are far-reaching and can affect bacterial metabolism, respiration, and regulation of colonization- and virulence-specific genes. Bacteria may utilize contact-dependent secretion systems and the associated effectors to rewire host cellular signaling pathways and favor persistence. Finally, adhesive structures themselves may act as extracellular effectors capable of altering host physiology in response to bacterial adhesion. Each of these mechanisms of host subversion, and how it is enabled by adhesion, will be discussed in more detail below. Much of the work addressing how adhesion facilitates host subversion and colonization is being done in pathogens. However, many of the principles discussed below equally apply to colonization by beneficial and commensal species.

### Adhesive structures and adhesion mechanisms determine the fate of bacteria–host interactions

The bacterial surface is a highly specialized organelle, and one of its key purposes is to facilitate adherence. The number of surface structures capable of mediating specific or nonspecific adhesion to surfaces is vast, and as such exceeds the scope of this review. However, an excellent overview of bacterial adhesins and adhesive surface structures was presented by Pizarro-Cerdaá and Cossart [1]. Depending on the biochemical identity of the adhesive structure, its role during colonization may vary: it may be to enable initial, weak, and nonspecific adhesion, by establishing hydrophobic interactions with the host surface, thereby overcoming the electrostatic repulsion between bacterial and host surface [2]. Other adhesins engage in highly specific interactions with host surface receptors, giving rise to high-affinity, stable interactions. The exact mechanism of adhesin–surface interaction is also a key determinant for the fate of the bacteria–host interaction: modular adhesins that engage multiple surface receptors, often in a co-operative manner, give rise to extremely high binding avidity. Examples include fibronectin-binding proteins, such as the *Staphylococus aureus* fibronectin-binding protein A or the *Borrelia burgdorferi* fibronectin-binding protein BBK32, which bind to fibronectin by forming a tandem β-zipper [3,4]. This zipper-like mechanism guarantees a productive interaction between bacterium and host cell and often initiates bacterial uptake by nonphagocytic cells [3]. Another, even more immediate way to trigger uptake by using a zipper-like mechanism is exemplified by the *Pseudomonas aeruginosa* surface lectin LecA, which forms a direct zipper with the host lipid membrane by binding the glycosphingolipid Gb3, thereby triggering membrane bending and facilitating invasion [5]. Another well-conserved adhesion mechanism is the interaction between bacterial lectins and glycoprotein or glycolipids on the host surface, which may form a catch bond. The molecular equivalent of a Chinese finger trap, the characteristic feature of this adhesive mechanism is that the dissociation rate decreases under tension. This is in stark contrast with many other adhesive interactions: even for extremely high-affinity, zipper-like mechanisms such as described above, physical force can easily disrupt the bacteria–host interaction, by distorting the binding epitopes, which ultimately leads to a structural mismatch between adhesin and host receptor domains and significantly weakens or altogether inhibits adhesion under tension [6]. The prototypical catch-bond forming adhesin is FimH, a lectin located on the tip of type I fimbriae that enables enhanced binding to host tissues under flow [7]. Since then, many other catch-bond forming adhesins have been identified, including ones containing van Willebrand factor domains, such as *P. aeruginosa* PilY [8]. The advantageous features of this mechanism include not only increased adhesion under physiological fluid shear, but also enable surface sampling under dynamic flow conditions and transduction of force across the bacterial cell envelope [8–10]. These examples are by far not exhaustive, but should serve to demonstrate that although bacterial adhesins are uniquely adapted to accomplish colonization of a specific niche, the mechanisms underpinning their function are well conserved across bacterial species.

### Surface sensing and adhesion can lead to adaptations in bacterial physiology that facilitate persistence

#### The role of mechanosensing in initiating adhesion

**Figure 1.**
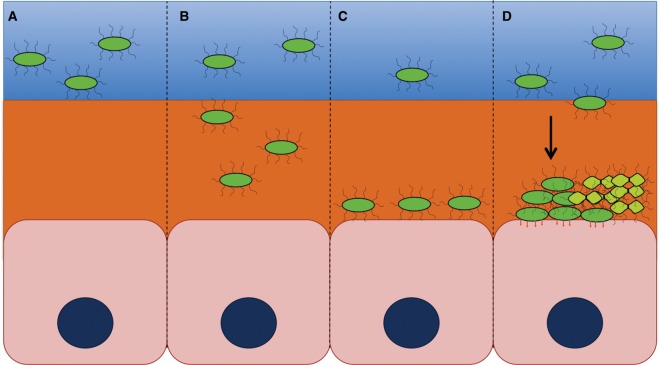
Surface sensing and adhesion as a multistep process. Through initial chemical sensing (**A**) and mechanosensing (**B**), bacterial cells are able to determine their proximity to a surface and regulate their physiology accordingly, including changes in metabolism, quorum sensing, and production of virulence factors. These changes following initial surface sensing can lead to reinforcement of the initial interaction (**C**). Reinforced attachment, together with the recruitment of additional planktonic bacteria from the liquid phase or proliferation of surface-bound bacteria, may lead to microcolony/biofilm formation (**D**) and bacterial persistence.

Prior to discussing the consequences of adhesion for colonization and persistence, it is important to define and distinguish the processes following on from the initial event, surface sensing. When a bacterium finds itself in close proximity of a surface, it may perceive the adjacent surface in many ways, including chemical sensing (i.e. sensing of specific chemical moieties on the surface) and/or mechanosensing (i.e. sensing of changes in physical forces related to the surface), which together are termed surface sensing (Figure 1). Mechanosensing is a complex function that depends on external force, affinity for the surface, and cell rigidity [11]. For example, *S. aureus* cell wall mutants with altered cell wall rigidity can display aberrant surface sensing, because a certain amount of ‘stiffness’ is required to efficiently transduce external forces across the bacterial envelope [12,13]. This work is particularly interesting, as it highlights the fact that mechanosensing is not limited to Gram-negative bacteria, but is a conserved feature also found in Gram-positive organisms, despite their drastically different cell wall architecture. Often mechanisms of chemical and mechanosensing are hard to dissect — e.g. deletion mutants of surface appendages may show altered behavior either because the specific chemical interaction is needed (i.e. they contribute to chemical sensing) or because this interaction is critical to achieve a threshold affinity for mechanoinduction (i.e. physical sensing). What further complicates the investigation is that the process of surface sensing can either lead to a productive interaction between bacterium and surface, i.e. initial adherence and continued surface sensing, or can be nonproductive (i.e. the bacterium gains enough distance to terminate surface sensing and remains in a planktonic state).

For the purpose of this review, adherence or surface attachment is defined as a specific interaction between an individual bacterium and a surface. As a consequence of surface sensing and initial adherence, physiological changes may trigger the recruitment of planktonic bacteria and favor interbacterial interactions between the surface-attached and the recruited bacterium. Equally, such interactions may arise due to proliferation of surface-bound bacteria. The outcome of such indirect surface associations can be the emergence of microcolony or biofilm communities (Figure 1D). The changes emerging as a consequence of direct adherence (bacterium–host interaction), as opposed to subsequent microcolony or biofilm formation (interbacterial interaction), are often difficult to dissect as they are interconnected and often progress in parallel. However, studies analyzing the changes in directly adherent cells versus adherent communities strongly suggest that these steps reflect defined stages of bacterial development and are accompanied by distinct physiological changes [14].

#### Bacterial appendages involved in surface sensing

The first cue perceived during surface sensing is thought to be an impairment of motility. In several systems studied to date, this is perceived by the bacterial flagella [15]. While the exact mechanistic details linking flagellar impairment and surface sensing have yet to be uncovered, and may vary as much as the flagellar systems themselves, a common theme seems to be that stator function and ion flux are required for a functional sensor [16]. For *Vibrio cholerae*, flagellar sensing remains controversial, but some reports suggest that flagellar mechanosensing in this species works by sensing changes in membrane potential in response to increased drag [17]. In *Salmonella* ssp., flagella are implicated in sensing surfaces by sensing wetness, rather than impaired motility [18].

In addition to flagella, which are implicated in early sensing, type I and type IV pili are associated with surface sensing upon initial adhesion. The requirements for pili-mediated sensing and subsequent signal transduction have mostly been studied in *Escherichia coli* and *P. aeruginosa*, but many mechanistic details still remain to be determined [19,20]. In *P. aeruginosa*, it has been known for some time that infection requires type IV pilus-associated adhesion [21]. The same study established that the minor pilin PilY and pilus retraction where required for this process, however, the mechanistic details linking pilus retraction to induction of key virulence factors required for the establishment of infection, including type III secretion, remained unknown for a long time. Siryaporn et al. recently demonstrated that surface contact changes pilus tension upon retraction, which modulates the interaction between PilA and PilJ and results in transcriptional changes including genes involved in type III secretion [8,22]. In contrast with *P. aeruginosa*, less is known regarding the pathway linking surface sensing to transcriptional responses in *E. coli*. Generally, the Cpx two-component system controls surface sensing and adhesion in *E. coli*, including lab strains, and it has been suggested that hydrophobicity and wettability of the surface are key features modulating this pathway [23].

#### The effects of surface sensing on bacterial physiology

**Figure 2.**
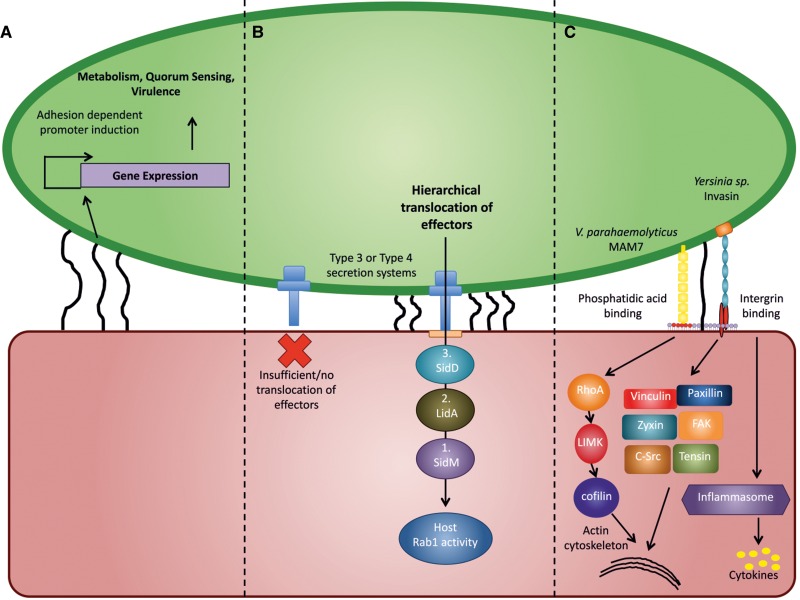
Several features of adhesion contribute to bacterial persistence. Attachment via bacterial adhesins and/or chemical sensing can alter bacterial cell physiology through regulation of different genes involved in metabolism, quorum sensing, motility, replication, and virulence, priming the bacteria for persistence (**A**). Stable adhesion is required to maintain hierarchical translocation of effectors into host cells, which is the basis of efficient manipulation of host cell signaling. Illustrated is the T4SS-dependent secretion of *L. pneumophila* effectors SidM, LidA, and SidD, which follow a strictly hierarchical order to sequentially modify host Rab1 activity on *Legionella*-containing vacuoles (**B**). Adhesins can directly affect host cell signaling by binding to surface receptors in the host cell membrane. Adhesin-induced receptor cross-talk or clustering of receptors in the host membrane can modify downstream signaling pathways within the host cell in a way that benefits bacterial persistence (**C**).

Adherence is also capable of modulating transcription in *E. coli* (Figure 2A). In enterohemorrhagic *E. coli*, attachment to host cells induces the locus of enterocyte effacement (LEE), a pathogenicity island encoding for a type III secretion system (T3SS) that contributes to disease severity. LEE induction levels were shown to be a function of both adhesion strength and shear force [24]. Enterotoxigenic *E. coli* (ETEC) and uropathogenic *E. coli* (UPEC) also modulate transcription in response to host cell attachment, and genes demonstrated to promote fitness during colonization in these studies included toxins and adhesins, as well as genes involved in iron transport for ETEC and UPEC, respectively [25,26]. Other species known to respond to surface adherence by triggering transcriptional changes include *Neisseria meningitidis*, *Caulobacter crescentus*, and *Trichomonas vaginalis*. Interestingly, not all of these changes are directly implicated in bacterial pathogenicity — often they affect global functions underpinning bacterial physiology, including DNA and RNA processing, genome stability, and bacterial metabolism [27–30]. In conclusion, if a bacterium adheres to a surface, no matter if this is a short-lived or prolonged interaction, this can be termed colonization. However, to achieve stable colonization (i.e. persistence), the adherent bacterium has to adapt to this niche in ways that protect it from immunological and physiological clearance. Consequently, physiological responses triggered by surface sensing include changes in metabolism, efflux, bacterial surface composition, and virulence factor production (Figure 2A). These changes can affect the outcome of colonization and determine the level of persistence, and are sustained by bacterial adherence [31–33]. The increasing commercialization of technology underpinning the investigation of surface sensing, such as microfluidic devices, atomic force microscopy, and optical traps, will allow us to — quite literally — push the envelope and our ability to further dissect mechanisms of and responses to bacterial surface sensing.

### Bacterial effector secretion and translocation require adherence

#### The importance of translocation hierarchy during pathogenesis

Many microbes capable of a host-associated lifestyle manipulate host cellular signaling to facilitate persistence. This is commonly achieved by the transfer of signaling effectors from the bacterial cell into the host cell's cytoplasm via contact-dependent secretion systems (Figure 2B). Effectors target a vast range of eukaryotic signaling hubs, and their activities are often aimed at fine-tuning innate immune signaling, chemotaxis, or phagocytic function, or at enabling persistence of bacteria in or on mucosal surfaces, for example by facilitating invasion, cell-to-cell spread, or blocking epithelial sloughing, among others [34,35].

Bacteria usually transfer a wide arsenal of effectors (ranging from a few to ∼300 different effectors in the case of *Legionella* [36]) through contact-dependent type III, type IV, or type VI secretion systems to target different aspects of host signaling. In doing so, the effectors are not all translocated simultaneously. Rather, they have to follow a stringent hierarchy to achieve a specific temporal profile which guarantees their activities are co-ordinated and can target a specific aspect of the host signaling network at the right time during infection. This is especially important because some effectors have synergistic or opposing activities [37–39]. This is the case with *Legionella pneumophila* effectors SidM and SidD, which act sequentially during the maturation of *Legionella*-containing vacuoles to first recruit and AMPylate, and later de-AMPylate the small GTPase Rab1 [37,38]. Enteropathogenic *E. coli* equally use sequentially delivered T3SS effector molecules to first activate filopodia formation, which aids the initial capture of bacteria, through translocation of Map, and later neutralize Map's Cdc42 targeting activity by Tir, which is also involved in the formation of pedestals, a more stable means of attachment [39]. In both cases, the temporal order of effector activity is essential to successful infection and can only be achieved if the bacterium remains stably attached to the targeted host cell throughout the translocation process (Figure 2B).

Studies elucidating the hierarchy and length of *Salmonella* pathogenicity island (SPI)-1 T3SS-mediated effector transfer in real time have demonstrated that the process takes more than 90 min to complete [40,41]. In *Salmonella enterica*, this intimate adhesion is facilitated by the SPI-4-encoded giant nonfimbrial adhesin SiiE. The SiiE protein contains 53 bacterial Ig (BIg) domains with lectin-like activity, which mediate extremely tight interactions with the apical side of polarized epithelial cells, and this intimate association is necessary for SPI-1 T3SS-mediated invasion [42]. Consistent with these studies, it has been shown that premature termination of the bacteria–host association during T3SS effector translocation renders the interaction nonproductive even if it has progressed for a considerable amount of time, and completely abolishes effector-mediated cytotoxicity on host cells [43].

#### Is specific adhesion required for effector translocation?

While adhesion is strictly required for efficient effector translocation, it is usually not required for effector production or even secretion [44]. In most cases, it is sufficient to maintain a high-affinity interaction, no matter the means. This means that often, partial loss of adhesin function can be tolerated and secretion activity is maintained, albeit at a lower level [45]. In *Pseudomonas*, for example, effector transfer can be maintained by different means of adhesion, and adhesins are interchangeable as long as the overall affinity is sufficiently high to maintain effector translocation. In *Yersinia*, YadA is more important than invasin with regard to effector translocation, due to the ability to bind to a broad range of host receptors on different cell types, thus ensuring high-affinity, shear-resistant interactions are maintained to initiate infection of different cell types [46,47]. Often, this is achieved by positive feedback between effector-mediated signaling and adhesion, which reinforces the initial adhesion to maintain sufficiently high affinity to ensure the process is not abrogated prematurely. This is the case for *Shigella flexneri*, where the T3SS effectors OspE1 and OspE2 mediate induced adherence to the colonic epithelium [48]. Another example is EHEC, where initial adherence to epithelial cells induces T3SS. The T3SS-mediated interaction between intimin and the translocated receptor, Tir, reinforces the interaction with host cells and further enhances T3SS translocation efficiency. In the absence of Tir, the interaction with the host surface is weakened and translocation efficiency drops [49].

In some cases, however, the mode of adhesion is important to adequately promote secretion. In the case of *Helicobacter pylori* T4SS, the host cell's pro-inflammatory response toward the adhesin Bap primes the cells to respond correctly toward the action of the T4SS effector CagA and thereby potentiates the function of the T4SS activity [50]. A further consideration is the constraint brought about by adhesion geometry, since adhesins protruding far from the surface may be suited to promote initial adherence, but may interfere with the activity of secretion systems. This has been described for the *Bartonella henselae* trimeric autotransporter adhesin BadA. BadA expression interferes with effector translocation by the VirB/D4 T4SS by increasing the distance between bacterial surface and host membrane, implying that the expression of specific adhesins and T4SS have to be differentially regulated to successfully infect [51].

#### The interplay between adhesion and secreted toxins

Finally, some consideration has to be given to the interplay between soluble toxins and adherence. In a way, secreted soluble toxins are often a more autonomous version of effectors — they still have effector domains, but are large and complex entities because in addition to the signaling targeting domain(s), they also have to encode the information for secretion, host binding, and translocation. There are examples where similar effector activities are achieved in a much more efficient way through contact-dependent secretion, compared with the toxin version [52]. But despite their ‘autonomy’, soluble toxins still require close proximity to the host because their local concentration is critical to their affinity for host receptors and hence, activity [53]. The importance of adhesion to toxin activity is underpinned by the observation that, like translocated effectors, toxins often reinforce bacterial adherence to enhance their own activity. The secreted ETEC toxin LT, for example, leads to increased presentation of receptors necessary for ETEC adherence on the host surface [54].

### Adhesins as direct effectors of host signaling

While adhesion is essential for the translocation of effectors, which facilitate bacterial persistence, it has emerged over the past few years that some adhesins fulfill a dual function during colonization and can themselves contribute to a microbe's repertoire of effector activities. Since adhesion and effects on host signaling are so interlinked, these two activities have been difficult to dissect genetically, using a reductionist approach — deletion of adhesins often impacts effector function by abrogating translocation efficiency. Rather, a minimalistic approach, studying adhesion and resulting signaling using either nonadhesive heterologous strains expressing adhesins [55], or purified adhesins reconstituted on a surface that geometrically resembles bacteria, has facilitated the analysis of effector activities mediated by adhesins [56]. The following are some examples to demonstrate the breadth of interactions and signaling pathways co-opted by adhesins. The topic was more comprehensively reviewed by Stones and Krachler [57].

#### Adhesins as effectors of integrin signaling

The integrin signaling axis was one of the first pathways described to be co-opted by bacterial adhesins. Integrins are surface receptors involved in a wide range of functions, including the regulation of cell–cell and cell–matrix interactions as well as inflammatory responses. Most notably, integrins are directly engaged by *Yersinia* invasin to initiate bacterial internalization, but this interaction also induces pro-inflammatory mucosal responses via activation of the NLRP3 inflammosome complex, which are beneficial for bacterial spread [58]. UPEC also subvert host integrins for invasion of bladder urothelial cells, and this is mediated by interactions between the FimH adhesin on the tip of type I pili and N-linked oligosaccharides on α3 and β1 integrins [59]. The gastric pathogen and carcinogen *H. pylori* also co-opt integrin signaling for delivery and activation of the type IV-secreted effector cytotoxin-associated gene A (CagA) within gastric epithelial cells, where it acts as an oncoprotein. In this case, the integrin α5β1 complex acts as a receptor for and is activated by CagL, an effector adhesin on the pilus surface. This activation results in CagA translocation and downstream-activation of focal adhesion kinase and Src, which is required for phosphorylation and activation of CagA [60].

A wide range of bacterial adhesins bind carcinoembryonic antigen (CEA) family proteins, an abundant family of glycoproteins on the apical surface of epithelial cells, as well as CEA-related cell adhesion molecules (CEACAMs). Binding of the prototypical CAECAM-binding adhesin, colony opacity associated (Opa) proteins from *Neisseria gonorrhoeae*, and other CEACAM-binding proteins of the Afa/Dr adhesin family increases integrin activity, thereby reinforcing adhesion of infected cells to the underlying substratum. Thus, this group of adhesins indirectly engages integrin signaling to prevent mucosal exfoliation and facilitate persistence within the host [61].

#### Rewiring of host signaling pathways by adhesins

In some cases, adhesins affect host signaling by providing the extracellular scaffolding to create novel signaling platforms. The oral pathogen *Porphyromonas gingivalis*, for example, uses fimbriae to cross-wire the Toll-like receptor TLR2 and the chemokine receptor 4 (CXCR4), which ultimately results in inhibition of TLR2-driven, pro-inflammatory responses and prolongs *P. gingivalis* survival [62]. The multivalent adhesion molecule (MAM) 7 adhesin of the food-borne pathogen *Vibrio parahaemolyticus* binds to the host membrane lipid phosphatidic acid with high affinity, thereby creating clusters of the lipid in the plasma membrane which act as signaling platforms for the activation of the small GTPase RhoA. Ultimately, this compromises cell–cell junction integrity and facilitates breaching of the epithelial barrier, as well as creating a larger surface area for the engagement of T3SS, thereby enhancing the efficiency of effector transfer [63,64].

These examples demonstrate that adhesins can play an important role as extracellular effectors, and their activities can promote invasion [37,38], fine-tune inflammatory responses [50,62], enhance the efficiency of contact-dependent secretion systems and associated effectors [50], and prevent cell sloughing [48,61]. The list of adhesion-mediated effector functions will likely become longer as research in this area intensifies. Adhesins are able to target receptor complexes with exquisitely high specificity, or even re-wire and create new pathways through signaling networks. Additionally, many adhesins are relatively easy to produce and more stable, compared with many other reagents used to interrogate eukaryotic signaling with such high specificity (e.g. antibodies). Through these beneficial features, adhesins will become, much like translocated bacterial effectors, indispensable tools to study host signaling pathways and help us gain novel insights, especially into surface receptor-mediated signaling pathways.

### Bacterial adhesion — an attractive therapeutic target?

As the above examples highlight, adhesion is strictly associated with bacterial persistence within the host and loss of adhesive features abrogates or completely abolishes long-term colonization in many cases. Adhesion, and specific adhesins, have thus become an attractive therapeutic target in the fight against infection and in the face of rising antimicrobial resistance, and many approaches to adhesion inhibition are being explored and developed (see ref. [65] for a recent review of approaches under development). Particularly, vaccines [66] and therapeutic antibodies targeting colonization [67] have progressed furthest through the drug development pipeline and, in a recent portfolio review, were identified as among the alternative approaches to antibiotics most likely to be translated into clinical use over the next decade [68].

#### Can bacteria bypass the need for adhesion and still persist?

While targeting adhesion will undoubtedly relieve selective pressure on antimicrobial resistance [69], it is less clear if this strategy may drive the selection for isolates that are capable of bypassing the requirement for adhesion, and still persist. If bacteria are in a planktonic state within the body, they will be removed by physical and immune clearance, unless they proliferate at a rate that exceeds the removal rate [70]. This is possible and occurs at sites that are nutrient rich, such as the intestinal lumen. It is thought that the host's provision of adhesin receptors provides a means of positive selection for a beneficial microbiota and can influence this principle, by supporting the persistence of slow-growing beneficial species by provision of specific attachment sites [71]. However, this selection can work both positively and negatively, if adhesion sites are provided in the form of renewable matrix that is turned over (e.g. mucus). Such sites, under some conditions, may select negatively against adhesive microbes and promote the persistence of a planktonic, luminal population [71]. We have to bear in mind that the consequence of adhesion inhibition is merely the displacement of bacteria into a planktonic state, not clearance *per se*. As such, the site of action, and potential of this site to support bacterial proliferation or promote clearance, is an important factor to consider when developing therapeutic strategies targeting adhesion.

Little is known about how displaced, planktonic bacterial populations would behave at most body sites. To close this gap in knowledge, displacement of bacteria and subsequent proliferation, physical and immune clearance and ultimately the fate of the microbe–host interaction, needs to be studied following adhesion inhibition *in vivo*. While such approaches may work well at sites with high natural clearance rates (e.g. bladder), it is hard to predict the outcome in niches with lower clearance (e.g. wounds) and with a well-established microbial community under the influence of host selection (e.g. intestinal tract) [70]. Thus, it is essential to collect experimental data regarding microbial behavior and host–microbe interactions at such sites, which will underpin our attempts to understand and predict the efficacy of therapies targeting infections at these sites. The limited studies undertaken to survey this have, however, shown up potential avenues to bacterial resistance to adhesion inhibition: an investigation of the efficacy of MAM-based adhesion inhibitors targeting multidrug-resistant bacterial infections have revealed a connection between the ability of a pathogen to adhere and mediate host cytotoxicity. While most investigated isolates show a strict correlation between adherence and cytotoxicity, some isolates were not as stringently dependent on adhesion to mediate host cell killing [72]. It will be important to further investigate the basis of this ability to bypass adhesion.

## Conclusions

While the fate and adaptation of bacteria under the pressure of adhesion inhibitors is unclear, it is clear that microbes have evolved strategies to try and bypass the stringent requirements of host attachment for pathogenesis. Examples include the packaging of toxins into outer membrane vesicles, which provide a means to overcome diffusive loss and enrich toxins at the site of action [73–75]. The ability to attach to other, host-associated bacteria instead of directly to the host, is another way of bypassing the requirement for host-directed adhesins. As such, biofilms, and especially polymicrobial biofilms, are an equally important area of investigation if we wish to understand the potential of adhesion inhibition as future therapies.

## Abbreviations

CagA: cytotoxin-associated gene A
CEA: carcinoembryonic antigen
CEACAMs: CEA-related cell adhesion molecules
ETEC: enterotoxigenic *E. coli*
LEE: locus of enterocyte effacement
MAM: multivalent adhesion molecule
SPI: *Salmonella* pathogenicity island
T3SS: type III secretion system
UPEC: uropathogenic *E. coli.*
